# CAR-based cellular therapy for refractory systemic lupus erythematosus: an overlap-controlled systematic review

**DOI:** 10.3389/fimmu.2026.1879668

**Published:** 2026-07-01

**Authors:** Sheng-Guang Li, Ruohan Yu, Lina Zhang, Jing Zhang, Yadan Zou, Ji Li, Ting Long, Yanfeng Zhang, Jing Pan

**Affiliations:** 1 Department of Rheumatology and Immunology, Peking University International Hospital, Beijing, China; 2 State Key Laboratory of Experimental Hematology, National Clinical Research Center for Blood Diseases, Haihe Laboratory of Cell Ecosystem, Institute of Hematology and Blood Diseases Hospital Chinese Academy of Medical Sciences and Peking Union Medical College, Tianjin, China; 3 State Key Laboratory of Experimental Hematology, Department of Hemato-oncology and Immunotherapy, Beijing GoBroad Hospital, Beijing, China

**Keywords:** CAR-NK, CAR-T, cellular therapy, lupus nephritis, systemic lupus erythematosus

## Abstract

**Objectives:**

To define the current evidence for CAR-based cellular therapy in relapsed or refractory systemic lupus erythematosus (SLE) by resolving overlapping reports and synthesising patient-level phenotype, safety and efficacy data. The primary purpose was evidence mapping with duplicate-count control; mechanistic immune-reset interpretation and platform differences were treated as secondary, hypothesis-generating questions rather than formal comparative-effectiveness analyses.

**Methods:**

PubMed, Embase and Web of Science were searched for reports published from January 2020 to March 2026. Eligible records described CAR-based cellular therapy in relapsed, refractory or organ-threatening SLE. Overlapping publication streams were collapsed into independent study units, and a deduplicated patient-level master dataset was constructed. Outcomes were summarised descriptively using conservative exact patient-level attribution, with endpoint evaluability reported separately from observed outcome proportions.

**Results:**

Twenty-one independent study units comprising 114 counted patients were included. The cohort was predominantly female (100/114, 87.7%), young (median age 32.0 years) and heavily pretreated, with renal involvement in 89 patients (78.1%). Autologous CD19 CAR-T and BCMA-containing platforms accounted for most treated patients, with additional allogeneic CAR-T and CAR-NK experience. Among exactly evaluable rows, clinical response was documented in 45 of 45 patients, DORIS remission in 13 of 15, SLE Responder Index-4 response in 20 of 20, renal improvement in five of five and drug-free remission in 28 of 58. These proportions should not be read as pooled response rates because exact endpoint denominators were small or incomplete for several outcomes. Cytokine release syndrome was usually low grade, and immune effector cell-associated neurotoxicity syndrome was uncommon. Severe inflammatory toxicities occurred in selected paediatric or highly inflammatory settings. Endpoint evaluability varied substantially across studies.

**Conclusion:**

CAR-based cellular therapy shows substantial promise as an immune-reset strategy for refractory SLE, particularly in renal-dominant disease. However, current evidence remains early, uncontrolled and incompletely reported. Sparse exactly evaluable denominators, publication bias, selective outcome reporting and short follow-up preclude definitive platform comparisons, supporting standardised prospective studies with harmonised efficacy, immune-reconstitution and toxicity endpoints.

**Systematic review registration:**

https://www.crd.york.ac.uk/prospero/, identifier CRD420261330006.

## Highlights

- CAR-based cellular therapy shows promising remission signals in refractory systemic lupus erythematosus.
- Overlap-controlled synthesis reduces duplicate counting in this rapidly expanding evidence base.
- Endpoint-specific proportions are descriptive only and remain limited by sparse exact evaluability.
- Future trials need harmonised efficacy, immune-reconstitution and immune-effector toxicity endpoints.

## Introduction

Systemic lupus erythematosus (SLE) is a prototypic systemic autoimmune disease characterised by loss of immune tolerance, pathogenic autoantibody production, immune-complex deposition, and heterogeneous multi-organ injury (1–6). Although outcomes have improved substantially over the past two decades, a clinically important subgroup of patients remains difficult to control, particularly those with lupus nephritis, haematological disease, neuropsychiatric involvement, or repeated relapses despite conventional immunosuppression and biologic therapy (2, 7, 8). Contemporary management is increasingly framed by treat-to-target principles, with remission and low disease activity recognised as preferred therapeutic goals; however, durable drug-free remission remains uncommon in routine practice (7–12).

This gap between disease suppression and true immune reset has provided the rationale for cellular immunotherapy (3, 13–15). Compared with antibody-based B-cell depletion, chimeric antigen receptor (CAR)-based therapy offers the possibility of deeper and potentially more durable elimination of autoreactive B-lineage cells, and in some platforms broader targeting of plasma-cell compartments through BCMA-directed or dual-target constructs (13–15). Early reports of anti-CD19 CAR-T therapy in refractory SLE, followed by expanding case series and phase 1 studies, have generated substantial interest by showing rapid clinical improvement, serological normalisation, and in some patients sustained remission after withdrawal of background immunosuppression (16–25).

However, the emerging literature is small, heterogeneous, and vulnerable to duplicate patient counting because initial index cases are often followed by expanded cohorts, long-term follow-up reports, or companion publications (15, 26–28). We therefore did an overlap-controlled systematic review with patient-level synthesis to define the current evidence base for CAR-based cellular therapy in relapsed or refractory SLE. The primary analytic purpose was to reconstruct the evidence architecture after overlap removal and to summarise phenotype, endpoint evaluability, safety and efficacy without duplicate counting. Mechanistic immune reset, off-the-shelf feasibility and target or platform differences were evaluated as secondary interpretive questions, not as formal comparative-effectiveness hypotheses.

## Methods

We did a systematic review with overlap-controlled patient-level synthesis of CAR-based cellular therapy in relapsed or refractory SLE. The protocol was prospectively registered in PROSPERO (CRD420261330006), and reporting was aligned with PRISMA 2020 principles (26). The analytic objective was twofold: first, to define the study-level evidence architecture after removal of duplicate publication streams; and second, to construct a deduplicated patient-level master dataset for descriptive synthesis of baseline phenotype, safety, and efficacy. A secondary interpretive objective was to discuss whether the observed clinical and serological patterns support an immune-reset hypothesis and to identify platform-specific questions for future trials. The review was not designed to compare CD19, BCMA, dual-target, allogeneic CAR-T, or CAR-NK platforms statistically.

We searched PubMed, Embase, and Web of Science for reports published from Jan 1, 2020, to March 2026. The search combined controlled vocabulary and free-text terms related to SLE, lupus nephritis, immune thrombocytopenia, CAR-T, CAR-NK, CD19, BCMA, CD22, and related cellular constructs or products. Database searches were supplemented by manual review of reference lists, forwards citation tracking, and review of linked or companion publications from known clinical programmes. Conference abstracts were eligible if they contained extractable clinical data.

Eligible records described clinical use of CAR-based cellular therapy in patients with confirmed SLE that was relapsed, refractory, highly active, or associated with major organ involvement despite previous standard treatment. Autologous and allogeneic CAR-T products were eligible, as were B-cell-directed or plasma-cell-directed CAR-NK platforms when used in a direct SLE clinical context. We included clinical trials, prospective or retrospective cohort studies, case series, conference abstracts with extractable data, and single-patient case reports. We excluded preclinical studies, manufacturing-only reports without clinical outcomes, duplicate reports without additional usable information, and reports in which CAR-based therapy could not be linked to an SLE population.

Because the literature is early and heterogeneous, the primary inclusive analytic dataset also retained one abstract-only paediatric report with extractable clinical information and one oncology-context anti-CD19 CAR-T case in which treatment was given for B-cell lymphoma in a patient with established SLE/antiphospholipid syndrome (29). These records were retained for completeness but flagged *a priori* for sensitivity interpretation.

**Figure 1:**
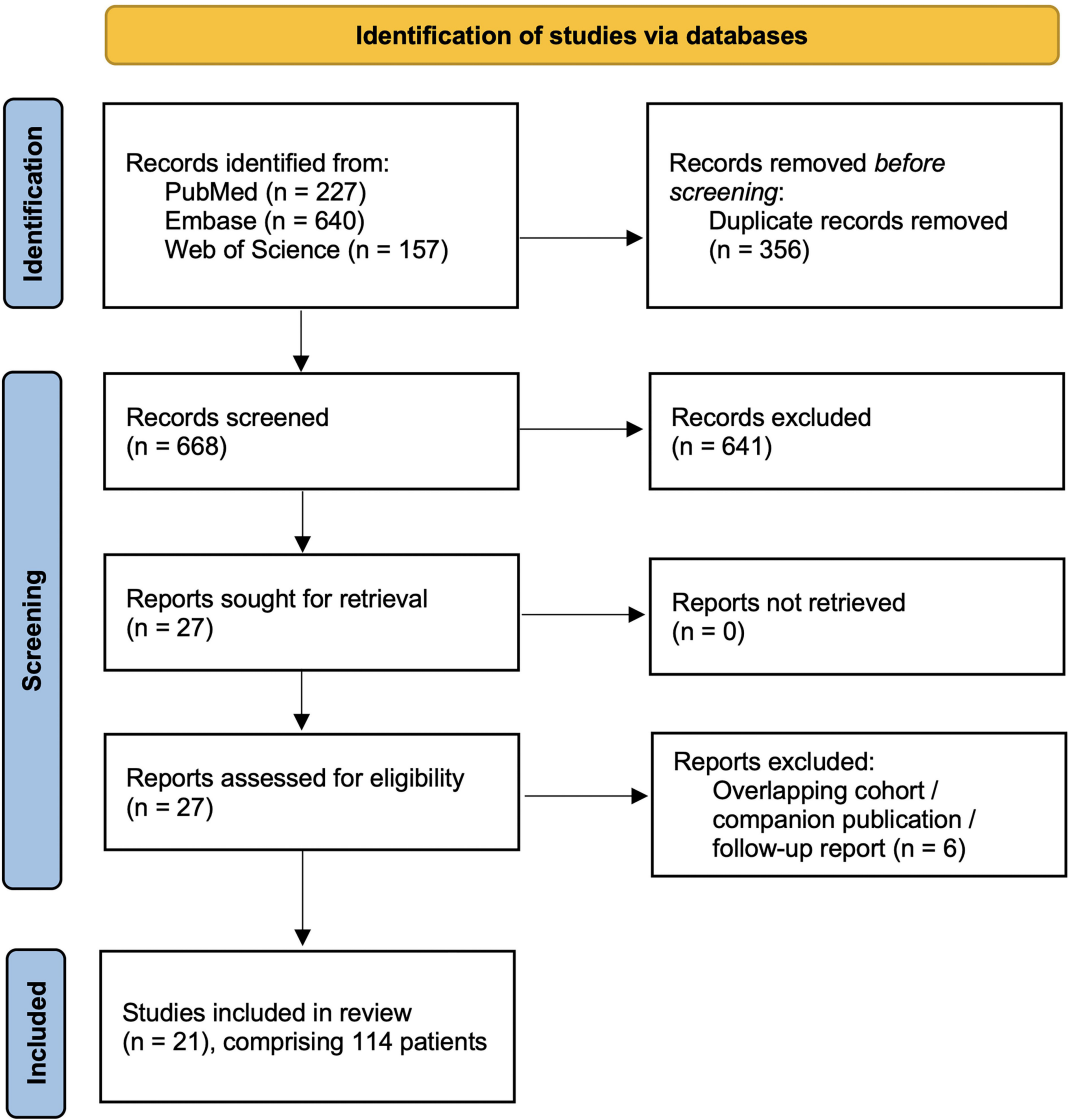
PRISMA flow diagram of study identification, screening, eligibility assessment, and inclusion. The figure shows the selection process for the systematic review. 1024 records were identified through database searches of PubMed, Embase, and Web of Science; 356 duplicates were removed before screening. After title and abstract screening, 27 reports underwent full-text assessment. Six reports were excluded because they represented overlapping cohorts, companion publications, or follow-up reports within the same publication stream. The final synthesis included 21 independent study units comprising 114 counted patients. Alt text: PRISMA flow diagram showing 1024 records identified, 356 duplicates removed, 27 full-text reports assessed, six overlapping reports excluded and 21 independent study units comprising 114 counted patients included.

A central methodological step was overlap resolution. When multiple publications clearly referred to the same patient stream or clinical programme, they were collapsed into a single independent study unit before patient counting. Typical overlap patterns included an index case followed by an expanded cohort, extended follow-up of a previously reported cohort, pregnancy or neonatal follow-up of an already reported patient, and parallel companion reports from the same product programme. For each publication stream, one primary counting source was selected; linked reports were used only to enrich follow-up or special outcomes. Ambiguous overlap was resolved conservatively in favour of avoiding duplicate counting. After full-text assessment, 21 independent study units were retained (**Figure 1**).

Data were extracted at two complementary levels: the study-unit level and the patient level. Study-level fields included country, publication year, design, sample size, cell source, platform, target, dose framework, follow-up, and broad efficacy and safety signals. Patient-level fields included age, sex, paediatric status, disease duration, baseline disease activity, organ involvement, lupus nephritis class when available, previous therapies, lymphodepletion, product or platform, acute toxicities, efficacy outcomes, and special events such as pregnancy or treatment withdrawal. One master row was created per treated individual. If the same patient appeared in more than one publication, all compatible information was merged into a single row and the most granular source was prioritised.

Baseline descriptors were age, sex, disease duration, disease activity, coded organ-domain involvement, and previous treatment exposure. Safety outcomes were any cytokine release syndrome (CRS), grade 3 or higher CRS, any immune effector cell-associated neurotoxicity syndrome (ICANS), grade 3 or higher ICANS, infection, severe infection, inflammatory complications including haemophagocytic lymphohistiocytosis, immune effector cell-associated HLH-like syndrome, or thrombotic microangiopathy, graft-versus-host disease, and treatment withdrawal or discontinuation. Efficacy outcomes were any clinical response, Definition of Remission in SLE (DORIS) remission, Lupus Low Disease Activity State (LLDAS), SLE Responder Index-4 (SRI-4) response, drug-free remission, and renal improvement (10–12, 30). Where source publications explicitly defined endpoints, source definitions were preserved; otherwise, terminology was harmonised only to the minimum extent needed for categorical coding.

Risk of bias was assessed at the study level with design-appropriate Joanna Briggs Institute critical appraisal tools for case reports, case series, and quasi-experimental or uncontrolled cohort studies, with methodological interpretation informed by guidance on synthesis of case reports and case series (31, 32). Domains considered included clarity of SLE diagnosis, clarity of CAR product and lymphodepletion description, consecutive or complete inclusion of treated patients, adequacy of baseline phenotype reporting, objective ascertainment of efficacy outcomes, adverse-event ascertainment and grading, follow-up duration, completeness of patient-level endpoint mapping, and clarity of confounding by concomitant or prior therapies. Because most included evidence was uncontrolled and outcome reporting was highly variable, risk-of-bias assessment was used to contextualise interpretability rather than to weight pooled estimates. No overall numeric quality score was calculated.

Data synthesis was prespecified at two levels: independent study units and deduplicated counted patients. Continuous variables were summarised as medians with IQRs, and categorical variables as counts and percentages. Baseline descriptive analyses used the full counted cohort (n=114), whereas outcome denominators varied according to endpoint evaluability.

**Figure 2:**
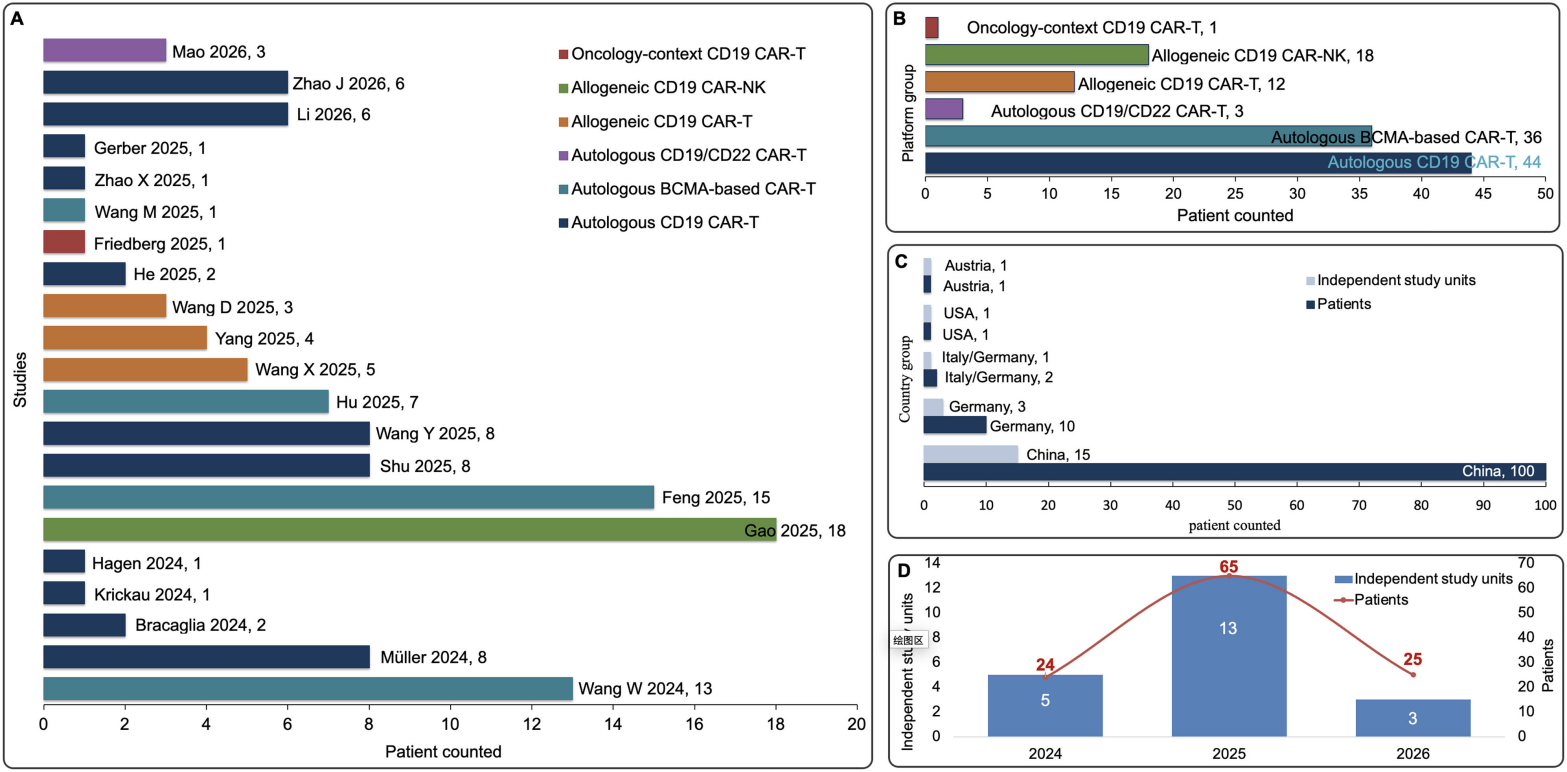
Evidence landscape and cohort architecture of the primary inclusive analytic dataset. **(A)** shows the counted contribution of each independent study unit, ordered by sample size and grouped by broad platform category. **(B)** shows the platform composition of the 114 counted patients. **(C)** shows the geographic distribution of the current evidence base, presented as counted patients and independent study units. **(D)** shows the distribution of counted patients by publication year. Counts are based on the deduplicated patient-level master dataset. Alt text: Evidence landscape figure showing study-unit contributions, platform composition, geographic distribution and publication-year distribution of 114 counted patients in the overlap-controlled dataset.

**Figure 3:**
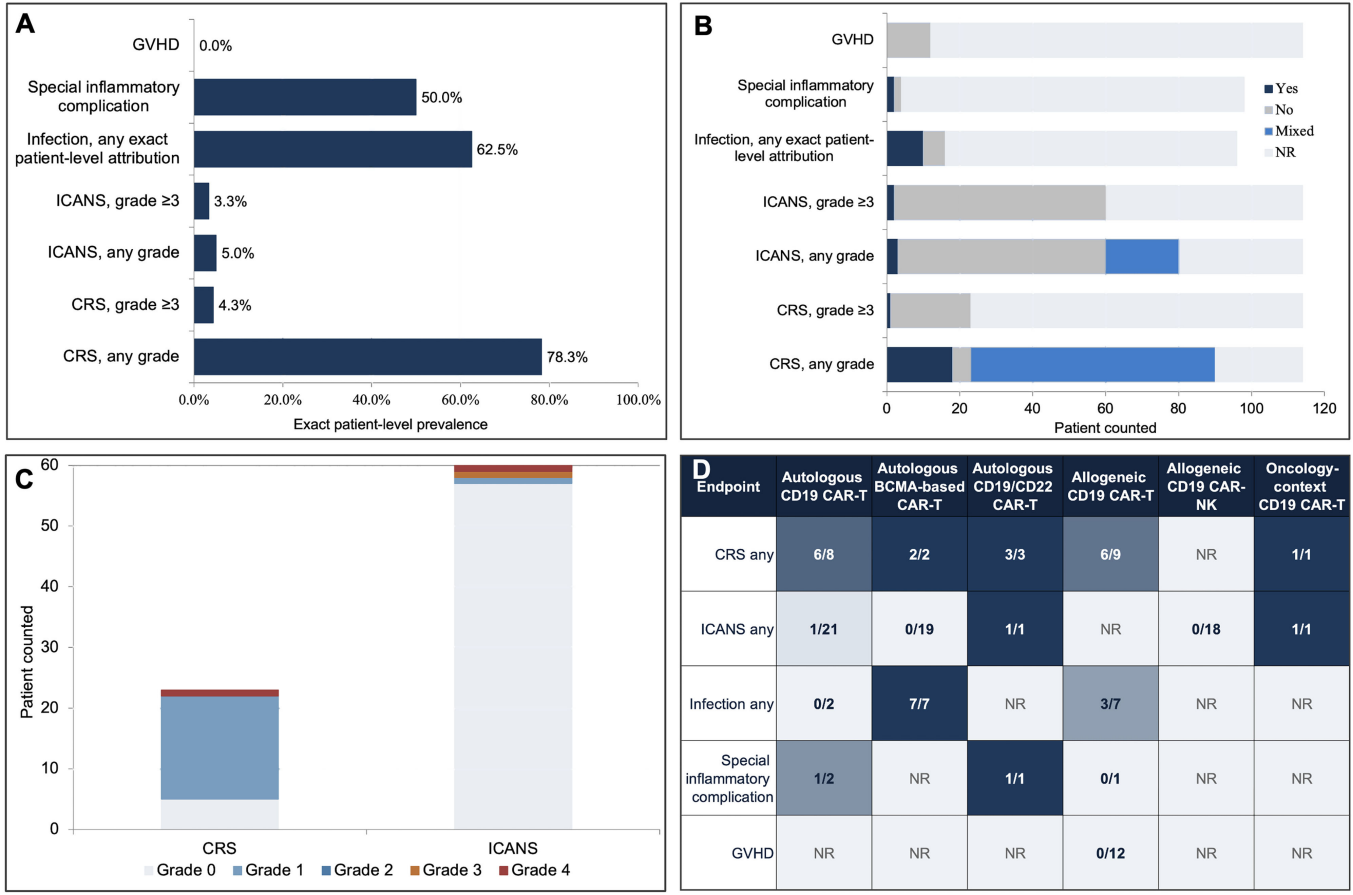
Safety outcomes and endpoint evaluability under conservative exact patient-level attribution. **(A)** shows the proportion of exactly attributable safety events amongst patients with exact endpoint evaluability. **(B)** shows endpoint evaluability across the counted cohort, separating exact yes or no attribution, mixed study-level reporting without patient-level mapping, and not reported. **(C)** shows the observed grade distribution for cytokine release syndrome and immune effector cell-associated neurotoxicity syndrome amongst exactly attributable rows. **(D)** shows platform-level safety signals, expressed as events amongst exactly evaluable rows. Exact attribution required either explicit patient-level reporting or a study-level universal positive or universal negative statement that could be conservatively mapped to all counted patients in that study unit. Mixed cohort-level summaries were retained in the evaluability analysis and were not reassigned to individual patients. Alt text: Safety outcome figure showing exact patient-level attribution and endpoint evaluability for cytokine release syndrome, ICANS, infection, severe inflammatory toxicity, graft-versus-host disease and treatment withdrawal.

**Figure 4:**
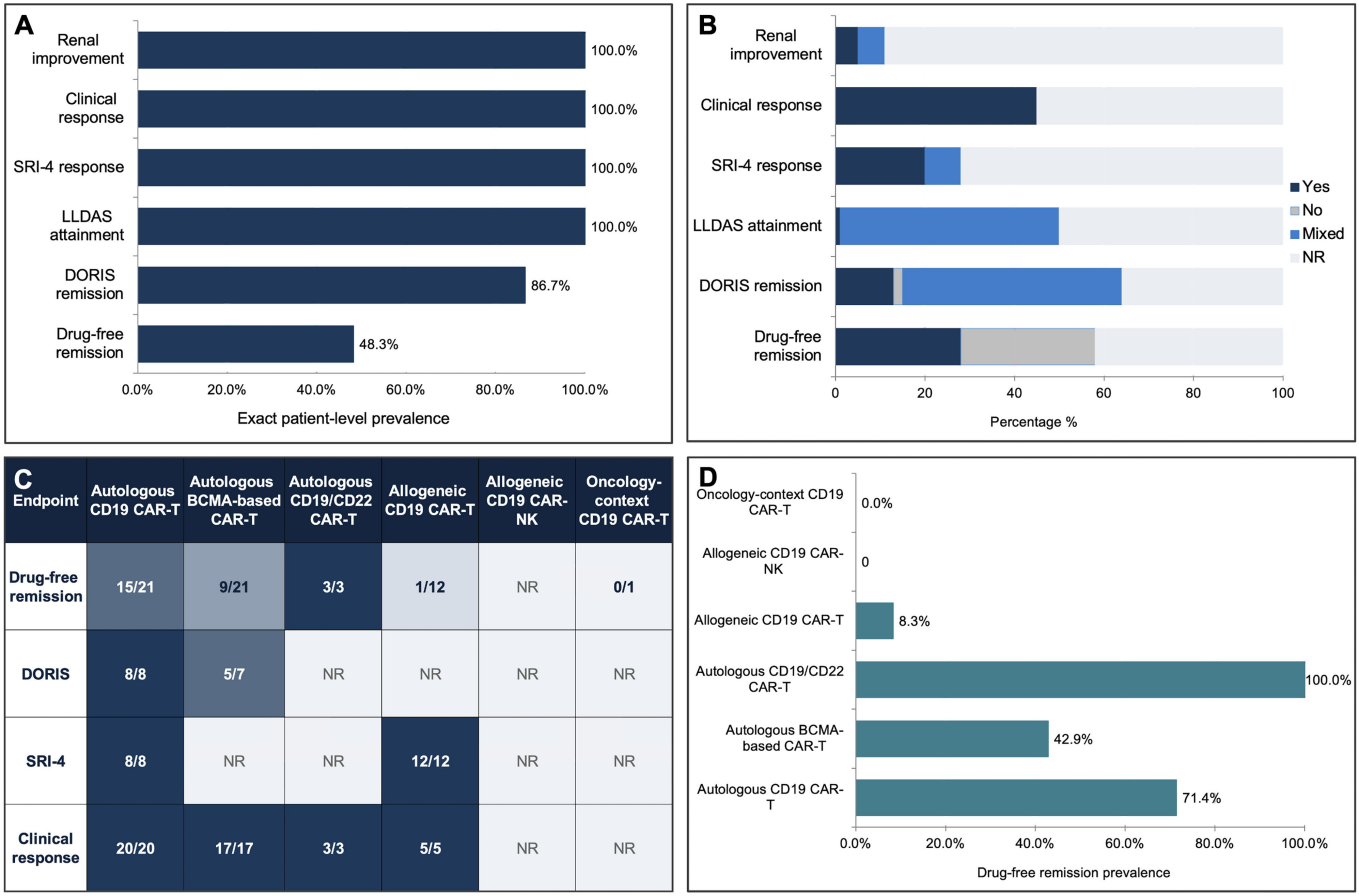
Efficacy outcomes and endpoint evaluability under conservative exact patient-level attribution. **(A)** shows the proportion of exactly attributable efficacy outcomes amongst patients with exact endpoint evaluability. **(B)** shows endpoint evaluability across the counted cohort, separating exact yes or no attribution, mixed study-level reporting without patient-level mapping, and not reported. **(C)** shows platform-level efficacy signals, expressed as positive outcomes amongst exactly evaluable rows. **(D)** focuses on drug-free remission, the most broadly attributable patient-level efficacy endpoint in the current dataset. Exact patient-level estimates should be interpreted as conservative summaries because several studies reported cohort-level outcomes without row-level mapping. Alt text: Efficacy outcome figure showing exact patient-level attribution and endpoint evaluability for clinical response, DORIS remission, SRI-4 response, renal improvement and drug-free remission across CAR-based platforms.

To minimise pseudo-precision, safety and efficacy outcomes were summarised with a conservative exact patient-level attribution rule. An endpoint was considered exactly attributable only when either a patient-level yes or no outcome was explicitly reported or a study-level universal positive or universal negative statement could be defensibly mapped to every counted patient in that study unit. Mixed cohort-level summaries, such as “three of seven patients had CRS” without individual mapping, were not redistributed to patient rows. Instead, each outcome field was classified as exact yes or no attribution, mixed study-level reporting, or not reported. This distinction was built directly into the presentation of results: **Table 1** and **Figure 2** describe the overlap-controlled study architecture, **Table 2** summarises the counted cohort, **Table 3** summarises risk-of-bias and reporting-quality domains, and **Figure 3** and **4** separately display exact outcome prevalence and endpoint evaluability.

**Table 1:** Characteristics of the 21 independent study units included in the primary inclusive analytic dataset.

Study	Country	Design	Patient number	Cell source	Product	Target	Lymphodepletion	Dose framework	Linked notes
Müller 2024 (19)	Germany	NEJM case series with follow-up	8	Autologous	MB.CART19.1/Erlangen anti-CD19 programme	CD19	Fludarabine + cyclophosphamide	1×10^6 CAR-T/kg	Primary counted SLE subset in Erlangen stream; earlier reports Mougiakakos 2021 and Mackensen 2022 not separately counted.
Krickau 2024	Germany	Lancet correspondence, single case	1	Autologous	Fresh anti-CD19 CAR-T	CD19	Renal-adjusted fludarabine + cyclophosphamide	1×10^6 CAR-T/kg	Independent single case.
Hagen 2024	Germany	Lancet correspondence, single case	1	Autologous	MB.CART19.1	CD19	Fludarabine + cyclophosphamide + dexamethasone prophylaxis	1×10^6 CAR-T/kg	Independent single case.
Bracaglia 2024	Italy/Germany	Conference abstract, 2-case report	2	Autologous	Prodigy-manufactured anti-CD19 CAR-T	CD19	Cyclophosphamide + fludarabine	1×10^6 CAR-T/kg	Independent abstract-level paediatric 2-case report.
He 2025	China	Letter/2-case paediatric series	2	Autologous	PrimeCAR	CD19	Cyclophosphamide + fludarabine	1×10^5 CAR-T/kg	Independent paediatric 2-case series.
Gerber 2025	USA	Short report, single-patient IND	1	Autologous	KYV-101	CD19	Fludarabine + cyclophosphamide	1×10^8 total cells	Independent single-patient IND report.
Hu 2025 (25)	China	Open-label single-arm LN trial	7	Autologous	Personalised anti-BCMA CAR-T	BCMA	Fludarabine + cyclophosphamide	2.5×10^6 cells/kg or 35×10^6 total cells	Independent BCMA-only LN trial.
Feng 2025	China	Nature Medicine phase 1 dose-escalation	15	Autologous	Co-infused anti-CD19 and anti-BCMA CAR-T	CD19 + BCMA	Fludarabine + cyclophosphamide	Study-specific dose-escalation	Independent dual-target co-infusion phase 1 study.
Shu 2025	China	Phase 1 relma-cel study	8	Autologous	Relmacabtagene autoleucel (relma-cel)	CD19	Fludarabine + cyclophosphamide	25/50/75/100×10^6 fixed dose	Independent Wuhan relma-cel cohort.
Wang Y 2025	China	Phase I relma-cel study	8	Autologous	Relmacabtagene autoleucel (relma-cel)	CD19	Fludarabine + cyclophosphamide	50/75/100×10^6 fixed dose	Independent Shanghai relma-cel cohort sharing trial registration but not duplicate.
Wang D 2025 (21)	China	Med single-arm pilot	3	Allogeneic	TyU19	CD19	Fludarabine + cyclophosphamide (reduced-intensity)	1×10^6 CAR+ cells/kg	Independent allogeneic TyU19 Med cohort.
Friedberg 2025	Austria	Oncology-context case report	1	Autologous	Axicabtagene ciloleucel	CD19	Oncology lymphodepletion	Standard axi-cel	Independent oncology-context APS/SLE case treated for lymphoma.
Gao 2025	China	Lancet first-in-human CAR-NK case series	18	Allogeneic CAR-NK	Cord/peripheral blood-derived CD19 CAR-NK	CD19	Fludarabine + cyclophosphamide	Schedule-/dose-escalation, three infusions/cycle	Independent allogeneic CAR-NK case series.
Li 2026	China	Med dose-escalation trial	6	Autologous	Anti-CD19 CAR-T (Juventas)	CD19	Fludarabine + cyclophosphamide	0.5×10^6 or 1×10^6 CAR-T/kg	Primary counted SLE-ITP cohort; earlier Li 2024 index case not separately counted.
Mao 2026 (24)	China	Kidney International Reports case series	3	Autologous	Bicistronic CD19/22 CAR-T	CD19 + CD22	Fludarabine + cyclophosphamide	1–10×10^6 CAR-T/kg	Independent bicistronic CD19/22 paediatric LN series.
Wang W 2024 (18)	China	Ann Rheum Dis phase 1 cCAR trial	13	Autologous	ICG318/BCMA-CD19 cCAR	BCMA + CD19	Cy/Flu for P1-2; cyclophosphamide alone for P3-13	3×10^6 cCAR/kg (P11 1.5×10^6/kg underdosed)	Primary counted Zhongshan BCMA-CD19 cCAR trial; long-term outcomes enriched by Hong 2026 follow-up.
Wang M 2025	China	Single translational overlap-syndrome case	1	Autologous	BCMA-CD19 cCAR	BCMA + CD19	Cyclophosphamide	3×10^6 cCAR/kg	Independent overlap-syndrome single case.
Wang X 2025	China	Nature Medicine phase 1 STAR-T trial	5	Allogeneic	YTS109 hypoimmune STAR-T	CD19	Fludarabine + cyclophosphamide	3×10^6 STAR+ cells/kg	Independent YTS109 STAR-T phase 1 cohort.
Yang 2025	China	Cell Research pilot letter	4	Allogeneic	TyU19	CD19	Reduced-intensity Flu/Cy in 3, none in 1	1×10^6 CAR+ cells/kg	Independent TyU19 pilot letter cohort from separate centre.
Zhao X 2025	China	Single case report	1	Autologous	Inaticabtagene autoleucel	CD19	Cyclophosphamide + fludarabine	30.15×10^6 total cells	Independent SLE-ITP single case.
Zhao J 2026	China	Ann Rheum Dis open-label pilot	6	Autologous	IM19	CD19	Fludarabine + cyclophosphamide	1×10^6/kg or flat 1×10^8	Independent IM19 anti-CD19 pilot cohort.

This table summarises the principal study-level characteristics of the 21 independent study units included in the primary inclusive analytic dataset. Variables include country, study design, counted sample size, cell source, platform, target, lymphodepletion, dose framework, and publication-linkage notes. Independent study units were defined after reconciliation of companion reports, follow-up reports, and partially overlapping publication streams to avoid duplicate patient counting. This table therefore presents the overlap-controlled study architecture used for the main synthesis.

**Table 2:** Baseline characteristics of the counted patient cohort overall and by broad platform group.

Metric	Overall	Autologous CD19 CAR-T	Autologous BCMA-based CAR-T	Autologous CD19/CD22 CAR-T	Allogeneic CD19 CAR-T	Allogeneic CD19 CAR-NK	Oncology-context CD19 CAR-T
Counted patients	114	44	36	3	12	18	1
Independent study units	21	11	4	1	3	1	1
Female sex	100/114 (87.7%)	39/44 (88.6%)	30/36 (83.3%)	2/3 (66.7%)	11/12 (91.7%)	17/18 (94.4%)	1/1 (100.0%)
Pediatric	12/114 (10.5%)	6/44 (13.6%)	2/36 (5.6%)	3/3 (100.0%)	1/12 (8.3%)	0/18 (0.0%)	0/1 (0.0%)
Age, years, median (IQR)	32.0 (23.0–37.0)	30.5 (21.0–35.2)	30.5 (25.2–36.0)	16.7 (15.2–16.9)	36.5 (23.0–38.8)	37.5 (32.0–39.8)	65.0 (65.0–65.0)
Disease duration, years, median (IQR)	9.0 (4.0–14.0)	7.0 (4.0–14.0)	8.5 (5.0–12.0)	1.1 (1.1–3.0)	11.5 (10.5–18.5)	10.5 (3.8–14.8)	23.0 (23.0–23.0)
Baseline SLEDAI/SELENA, median (IQR)	12.0 (10.0–16.0)	12.0 (8.5–17.0)	12.0 (8.0–16.0)	12.0 (10.0–16.0)	16.5 (14.0–20.0)	12.0 (10.0–14.0)	NR
Reported follow-up, months, median (IQR)	12.0 (6.0–15.0)	12.0 (7.0–12.0)	24.0 (11.2–30.0)	12.0 (10.5–13.5)	6.0 (6.0–6.0)	NR	NR
Lupus nephritis	89/114 (78.1%)	41/44 (93.2%)	28/36 (77.8%)	3/3 (100.0%)	8/12 (66.7%)	8/18 (44.4%)	1/1 (100.0%)
Multi-organ involvement (≥3 coded organ domains)	59/114 (51.8%)	34/44 (77.3%)	11/36 (30.6%)	1/3 (33.3%)	9/12 (75.0%)	4/18 (22.2%)	0/1 (0.0%)
High completeness rows	35/114 (30.7%)	4/44 (9.1%)	19/36 (52.8%)	3/3 (100.0%)	8/12 (66.7%)	0/18 (0.0%)	1/1 (100.0%)

This table summarises the baseline characteristics of the 114 counted patients in the primary inclusive analytic dataset, overall and stratified by broad platform group. Variables include demographic characteristics, age, disease duration, baseline disease activity, follow-up duration, renal involvement, multisystem disease burden, and completeness of patient-level reporting. Data are median (IQR) or n/N (%), unless otherwise specified. Denominators vary for some variables because reporting completeness differed across study units.

**Table 3:** Risk-of-bias and reporting-quality assessment across the overlap-controlled evidence base.

Domain	Main assessment findings	Direction of bias or limitation	Interpretive implication
Study design and controls	No included study was randomised or controlled; most were case reports, case series, letters, conference abstracts or single-arm early-phase cohorts.	High risk of selection bias and confounding by indication.	Signals support feasibility and hypothesis generation but not comparative effectiveness.
Patient selection	SLE diagnosis and refractory phenotype were usually clear, but consecutive enrolment and screening denominators were inconsistently reported.	Potential enrichment for severe, highly selected or successfully treated patients.	Generalisation to broader SLE or earlier disease populations remains uncertain.
Intervention ascertainment	CAR target, cell source and lymphodepletion were usually described; manufacturing details, washout periods and bridging therapy were variably reported.	Confounding by product differences and previous or concomitant therapy.	Target and platform comparisons must remain qualitative.
Outcome definitions	DORIS, SRI-4, renal response, LLDAS and drug-free remission definitions differed across reports or were incompletely mapped to patient rows.	Selective outcome reporting and endpoint heterogeneity.	Endpoint-specific proportions are descriptive and denominator-limited.
Safety reporting	CRS and ICANS were more consistently reported than infection, hypogammaglobulinaemia, delayed cytopenia, immune reconstitution or vaccine response.	Under-ascertainment of delayed or non-acute toxicity.	Long-term immune-safety conclusions are premature.
Follow-up	Median reported follow-up was approximately 12 months, with longer observation in selected cohorts and shorter follow-up in several off-the-shelf reports.	Durability and late safety may be overestimated or incompletely observed.	Prospective longitudinal follow-up is required.
Patient-level completeness	Exact patient-level attribution was feasible for only selected endpoints; mixed study-level reporting was common.	Risk of pseudo-precision if cohort-level events are redistributed to patients.	The exact-attribution rule intentionally yields smaller but more credible denominators.
Publication and reporting bias	The literature is dominated by early positive experiences, small cohorts and abstracts; unpublished failures cannot be assessed.	Likely preferential dissemination of successful remission cases.	Observed high response proportions should not be interpreted as unbiased absolute response rates.

This table summarises design-level limitations and reporting-quality domains used to interpret the included case reports, case series, single-arm studies, conference abstracts and early-phase cohorts. The purpose of this assessment was to contextualise certainty rather than to generate a weighted pooled estimate.

No formal between-platform hypothesis testing was done because the evidence base was non-comparative, sample sizes were small, and patient-level completeness differed substantially across platforms and endpoints. For the same reason, we did not undertake a pooled meta-analysis in the present manuscript, and funnel-plot assessment of publication bias was not appropriate. Publication bias and selective outcome reporting were therefore evaluated qualitatively by considering study design, abstract-only reporting, endpoint completeness, and the likelihood that early successful experiences are preferentially disseminated (33, 34). All descriptive summaries and figure-ready datasets were generated from the patient-level master workbook using spreadsheet-based curation and Python-assisted analyses.

## Results

The database search identified 1024 records, of which 356 duplicates were removed before screening. After title and abstract review, 27 reports underwent full-text assessment. Six reports were excluded because they represented overlapping cohorts, companion publications, or follow-up reports within the same publication stream. The final overlap-controlled synthesis therefore comprised 21 independent study units, including 114 counted patients treated with CAR-based cellular therapy for relapsed or refractory SLE or closely related high-relevance SLE phenotypes (**Figure 1**; **Table 1**).

The evidence base expanded rapidly over a short period. Most counted patients came from Chinese cohorts (100 of 114, 87.7%), with additional contributions from Germany, mixed Italy and Germany records, and single-patient reports from the USA and Austria. By publication year, counted patients increased from 24 in 2024 to 65 in 2025, followed by 25 in 2026, indicating rapid clinical translation across the most recent 3 years (**Figure 2**). Autologous platforms predominated: 44 of 114 patients (38.6%) received autologous CD19 CAR-T, 36 (31.6%) received autologous BCMA-containing products, three (2.6%) received autologous CD19/CD22 CAR-T, 12 (10.5%) received allogeneic CD19 CAR-T, 18 (15.8%) received allogeneic CD19 CAR-NK, and one patient (0.9%) received oncology-context anti-CD19 CAR-T. Across the 21 independent study units, the largest contributors were Gao 2025 (n=18), Feng 2025 (n=15), Wang W 2024 (n=13), Wang Y 2025 (n=8), Shu 2025 (n=8), and Hu 2025 (n=7; **Table 1****;** **Figure 2**).

Baseline characteristics are summarised in **Table 2**. The counted cohort was predominantly female (100 of 114, 87.7%) and relatively young, with a median age of 32.0 years (IQR 23.0-37.0), median disease duration of 9.0 years (4.0-14.0), and median baseline SLEDAI or SELENA-SLEDAI score of 12.0 (10.0-16.0). Twelve patients (10.5%) were paediatric. Among rows with available follow-up duration, median follow-up was 12.0 months (6.0-15.0), although longer follow-up was available in selected dual-target cohorts. Renal disease was the dominant phenotype: 89 of 114 patients (78.1%) had lupus nephritis or coded renal involvement, including all paediatric patients. Skin or mucosal involvement was reported in 66 patients (57.9%), musculoskeletal involvement in 47 (41.2%), and haematological involvement in 43 (37.7%). Overall, 59 of 114 patients (51.8%) had at least three coded organ domains, consistent with a heavily burdened multisystem population. Previous treatment exposure was extensive: 106 of 114 patients (93.0%) had received glucocorticoids, 87 (76.3%) hydroxychloroquine, 72 (63.2%) mycophenolate mofetil, 61 (53.5%) cyclophosphamide, and 60 (52.6%) a calcineurin inhibitor; prior exposure to belimumab, telitacicept, and rituximab was also common.

Risk-of-bias and reporting-quality findings are summarised in **Table 3**. The principal limitation was study design: no included study was randomised, and most evidence came from case reports, case series, letters, conference abstracts, or single-arm early-phase cohorts. SLE diagnosis and CAR product description were generally clear, but consecutive inclusion, prospective endpoint definition, adverse-event grading, immune-reconstitution monitoring and long-term follow-up were inconsistently reported. Patient-level efficacy mapping was substantially more complete for some autologous CD19 and BCMA-containing cohorts than for several off-the-shelf or abstract-level reports. Overall, the evidence supports feasibility and strong early clinical signals, but it remains at serious risk of selection bias, selective outcome reporting, incomplete adverse-event ascertainment and confounding by prior or concomitant immunosuppression.

Safety outcomes are shown in **Figure 3**. Under conservative exact patient-level attribution, CRS of any grade occurred in 18 of 23 exactly evaluable patients (78.3%), whereas grade 3 or higher CRS was reported in one of 23 (4.3%). ICANS occurred in three of 60 exactly evaluable patients (5.0%), including grade 3 or higher ICANS in two of 60 (3.3%). Infection of any type was exactly attributable in 10 of 16 evaluable patients (62.5%), but the denominator for severe infection was sparse, with three of three exactly evaluable rows documenting grade 3–4 or hospitalisation-level infectious events. Hyperinflammatory complications, including HLH-like, IEC-HS-like, or thrombotic microangiopathy-like events, were exactly attributable in two of four evaluable rows. These denominators are small and should be interpreted as descriptive amongst reported rows rather than as stable safety rates. No graft-versus-host disease was observed amongst 12 exactly evaluable allogeneic CAR-T recipients, and one study-level treatment withdrawal was documented because of unresolved thrombocytopenia requiring rescue immunosuppression.

Endpoint evaluability varied substantially and is itself an important finding (**Figure 3**). Exact yes or no attribution was available for only 23 of 114 rows (20.2%) for CRS, 60 (52.6%) for ICANS, 16 (14.0%) for infection, and 12 (10.5%) for graft-versus-host disease. Mixed study-level safety reporting without patient-level mapping was especially common for CRS (67 of 114, 58.8%) and special inflammatory toxicities (16 of 114, 14.0%), whereas infection and graft-versus-host disease were frequently not reported. Acute cytokine toxicity therefore appeared usually low grade when adequately described, but patient-level prevalence estimates for less consistently reported toxicities, particularly infection and delayed immune complications, should be interpreted with caution.

Efficacy outcomes are shown in **Figure 4**. In the subset permitting exact attribution, patient-specific clinical response was documented in all 45 of 45 evaluable rows. DORIS remission occurred in 13 of 15 exactly evaluable patients (86.7%), SRI-4 response in 20 of 20 (100%), and renal improvement in five of five (100%). Drug-free remission was the most broadly attributable efficacy endpoint and was documented in 28 of 58 exactly evaluable patients (48.3%). These results indicate a consistent favourable direction of effect amongst evaluable patients, but the small exact denominators for DORIS remission, SRI-4 response and renal improvement preclude robust rate estimation. By contrast, exact patient-level attribution for LLDAS was available in only one row, although several cohort reports described additional LLDAS attainment at study level without sufficient mapping to individual patients.

Across platform groups, the direction of effect was generally favourable, but the amount of attributable evidence differed. Autologous CD19 CAR-T contributed the largest exactly evaluable efficacy base, with drug-free remission documented in 15 of 21 evaluable rows, DORIS remission in eight of eight, SRI-4 response in eight of eight, and clinical response in 20 of 20. Autologous BCMA-containing platforms showed drug-free remission in nine of 21 evaluable rows and DORIS remission in five of seven. The small bicistronic CD19/CD22 paediatric series showed drug-free remission in three of three and clinical response in three of three, but also the strongest exact inflammatory toxicity signal. Allogeneic CD19 CAR-T studies showed SRI-4 response in 12 of 12 exactly evaluable rows with low neurotoxicity and no graft-versus-host disease, whereas patient-level efficacy attribution remained limited for CAR-NK because most reports used cohort-level rather than row-specific outcome reporting. These platform-level observations should be interpreted as hypothesis-generating rather than comparative.

Overall, the overlap-controlled master synthesis depicts a rapidly expanding but methodologically uneven evidence base. The current counted cohort consists mainly of young women with heavily pretreated, renal-dominant, multisystem refractory SLE. Within the subset allowing conservative patient-level attribution, remission and response signals were strong and acute inflammatory toxicity was usually manageable, but the interpretability of several endpoints remained constrained by incomplete or mixed reporting across study units.

## Discussion

Our overlap-controlled systematic review suggests that CAR-based cellular therapy in relapsed or refractory SLE has moved beyond isolated proof-of-concept rescue and is now better understood as an emerging therapeutic class (14–25, 27, 28, 35–42). The signal is not confined to one centre, one construct, or one publication stream. Since the first anti-CD19 CAR-T reports in SLE, the literature has expanded to include larger autologous CD19 experiences, BCMA-containing and dual-target strategies, allogeneic CD19 CAR-T products, allogeneic CAR-NK platforms, and early *in vivo* CAR-T approaches (16–25, 27, 28, 41, 42). Taken together, these studies indicate that the field is transitioning from anecdotal innovation to an early but coherent clinical evidence base. What remains unresolved is not whether the approach can work in selected patients, but in whom, with which target architecture, under which safety-monitoring framework, and at what point in the disease course it should be deployed. The present review therefore evaluates immune-reset plausibility and platform feasibility descriptively; it does not establish comparative efficacy between platforms.

A central message of this review is that the therapeutic effect is best interpreted through convergence across domains rather than through any single endpoint. In the available SLE studies, the most convincing pattern is the repeated alignment of rapid B-cell depletion, reduction in disease-activity scores, serological improvement, and tapering or discontinuation of background immunosuppression (17–19, 23, 24). This multidomain concordance matters in refractory SLE, in which single biomarker changes can be difficult to interpret and single clinical improvements can be confounded by concomitant glucocorticoid exposure. The immune-reset concept has become more biologically plausible because CD19 CAR-T treatment in autoimmune disease has now been linked not only to peripheral B-cell depletion but also to deep depletion of B cells in lymphoid and non-lymphoid tissues, with re-emergent B-cell compartments enriched for naive phenotypes (13, 15, 19, 43). That observation helps explain why remission can outlast the period of absolute peripheral B-cell aplasia.

The renal signal deserves particular emphasis. In our synthesis, renal involvement dominated the counted cohort, which is consistent with the fact that refractory lupus nephritis remains one of the clearest clinical settings in which an immune-reset strategy is attractive (8, 18, 24, 25). Early clinical data suggest that CAR-based therapy can be followed by sharp reductions in proteinuria, improvement in serological activity, and major reductions in overall disease activity, including in patients with nephritis-heavy phenotypes (18, 24, 25). At the same time, this review argues against an overly simplistic interpretation of renal response. CAR-based therapy is plausibly most effective against active immune-mediated renal injury; residual renal abnormality after systemic remission may reflect chronic structural damage rather than persistent immunological failure (8, 18, 25). For that reason, future lupus nephritis trials should stratify patients by baseline chronicity and, where feasible, incorporate histological or imaging-supported renal endpoints rather than relying exclusively on proteinuria-based readouts.

Target selection is likely to be one of the decisive questions for the next phase of the field. CD19-directed therapy established the concept, but it may not be the whole answer for every refractory SLE phenotype (14–18). CD19 is expressed across much of the B-cell lineage, including early B cells, mature B cells and many plasmablasts, and CD19 CAR-T may therefore affect not only autoantibody precursors but also B-cell antigen presentation, cytokine production and T-cell co-stimulation. However, long-lived plasma cells can reside in CD19-negative compartments, providing a biological rationale for approaches that target plasma-cell survival pathways more directly (13). BCMA is preferentially expressed on plasmablasts and plasma cells and is embedded in the BAFF/APRIL survival axis; BCMA-directed or BCMA-containing platforms therefore test a different mechanistic hypothesis: that some antibody-driven or nephritis-dominant SLE phenotypes require broader depletion extending beyond the CD19-positive B-lineage compartment to achieve a deeper serological reset (18, 25, 44, 45). This rationale is attractive, but it also raises the possibility of more pronounced hypogammaglobulinaemia or delayed infection risk if protective plasma-cell compartments are substantially affected.

The distinction between CD19-only, BCMA-only and CD19/BCMA compound approaches is therefore mechanistic as well as clinical. CD19-only therapy may be sufficient when disease is maintained primarily by autoreactive B-cell and plasmablast turnover and when reconstitution towards a naive B-cell repertoire can restore tolerance. BCMA-only therapy may be most rational when persistent autoantibody production from plasma-cell compartments is central, although BCMA-only targeting may spare upstream autoreactive B cells capable of regenerating the response. CD19/BCMA compound CAR-T or co-infused dual products attempt to close both gaps by depleting B-cell precursors and antibody-secreting compartments simultaneously (18). The promising results reported with BCMA-CD19 compound CAR-T constructs are therefore important not merely as incremental engineering but as a test of whether combined B-cell and plasma-cell targeting can deepen clinical and serological reset. Current data do not prove superiority of any target strategy; they define the trial questions that should now be tested prospectively.

Emerging B-cell targets beyond CD19 and BCMA may eventually permit more precise immune editing and therefore merit consideration. CD22 targeting extends B-lineage coverage and is already used in haematological CAR development; the paediatric CD19/CD22 lupus nephritis experience suggests feasibility but also illustrates that broader targeting does not eliminate inflammatory risk (24). BAFF-axis strategies are biologically relevant because BAFF, APRIL and their receptors BAFF-R, TACI and BCMA regulate transitional, mature, memory and plasma-cell survival, and BAFF/APRIL inhibition is already clinically relevant in SLE through belimumab and telitacicept (44–48). BAFF-R CAR-T has been developed in B-cell malignancies and is conceptually attractive for B-cell diseases, but direct clinical evidence in SLE remains too limited for quantitative integration with the CD19 and BCMA SLE datasets (49). Other antigen-restricted approaches, including autoreactive B-cell receptor or IGHV4-34-directed concepts, aim to spare the protective B-cell repertoire whilst eliminating pathogenic clones; plasma-cell targets such as CD38 or SLAMF7 are also biologically plausible, as illustrated by antibody-based plasma-cell depletion in refractory SLE, but on-target effects on protective humoral immunity remain a concern (50). At present, these platforms should be viewed as adjacent, preclinical, or very early translational strategies rather than evidence-equivalent alternatives to CD19 or BCMA clinical CAR datasets.

Safety should be interpreted with equal caution. Across the available literature, CRS in autoimmune disease appears generally milder than that seen in oncology CAR-T practice, and ICANS remains uncommon, which is biologically plausible in a lower target-burden inflammatory setting (17–19, 51, 52). However, our review also shows why reassuring averages can be misleading. Severe inflammatory toxicities did occur, and when they did, they tended to cluster in recognisable contexts, including paediatric lupus nephritis and highly inflammatory disease states (24, 25). The recent paediatric bicistronic CD19/CD22 lupus nephritis series is especially instructive: whilst remission was achieved, one patient developed grade 4 CRS with HLH and thrombotic microangiopathy (24). This argues strongly for using standardised ASTCT-based toxicity reporting across future autoimmune CAR studies and for separately capturing IEC-HS-like events rather than subsuming them under generic serious adverse events (51, 53). In this setting, safety is not a single class effect; it is likely phenotype-dependent, product-dependent, and possibly dose or schedule-dependent.

Long-term safety will probably become as important as acute CRS and ICANS in autoimmune CAR development. Prolonged hypogammaglobulinaemia, delayed bacterial or viral infections, late cytopenias, impaired vaccine responses and atypical immune reconstitution are well recognised after oncology CAR-T, particularly after B-cell or plasma-cell targeting (54–56). Autoimmune patients may have additional risk modifiers, including chronic glucocorticoid exposure, nephrotic-range proteinuria, complement abnormalities, previous cyclophosphamide or mycophenolate exposure, prior B-cell-directed biologics and baseline lymphopenia. Future SLE CAR studies should therefore report serial IgG, IgA and IgM levels, B-cell and plasma-cell reconstitution phenotypes, T-cell recovery, vaccine antibody titres when relevant, antimicrobial prophylaxis, immunoglobulin replacement, infection severity and pathogen-specific diagnoses. Without these data, early remission may be overvalued relative to delayed immune cost.

Prior therapies are another important source of confounding and should be captured more systematically. Rituximab, belimumab, telitacicept, cyclophosphamide, mycophenolate, calcineurin inhibitors and glucocorticoids can influence baseline B-cell and plasma-cell composition, target burden, T-cell fitness, manufacturing success, CAR expansion, infection risk and the apparent ability to stop background immunosuppression (46–48, 57, 58). Nonresponse to rituximab should not be assumed to predict nonresponse to CD19 CAR-T, because CD20-directed antibodies incompletely deplete tissue B cells and do not target CD20-negative plasmablasts or plasma cells, whereas CD19 CAR-T may achieve deeper tissue penetration and different effector mechanisms (17, 19, 43, 57, 58). Conversely, recent BAFF/APRIL blockade may alter B-cell reconstitution after CAR therapy and could theoretically reduce autoreactive rebound, but it could also confound attribution of remission or infection risk. Future trials should prespecify washout periods, document timing of prior biologics, stratify by prior B-cell or plasma-cell-directed therapy, and report whether remission persists after complete withdrawal of conventional immunosuppression.

The emergence of off-the-shelf platforms is one of the most important translational developments since the initial SLE CAR-T reports. Autologous products have shown remarkable efficacy, but they are constrained by manufacturing time, patient T-cell fitness, access, cost, and the clinical risk of waiting through a washout period in unstable disease (15, 20–23, 27, 28, 41, 42). Allogeneic CD19 CAR-T and CAR-NK approaches are designed to address exactly those bottlenecks (20–23, 41, 42, 59, 60). Early studies now suggest that these products can achieve profound B-cell depletion with acceptable short-term safety, whilst CAR-NK therapy may offer a particularly favourable acute toxicity profile (20–23, 41, 42, 60). At the same time, equivalence should not be assumed. Follow-up is shorter in several off-the-shelf studies, exact patient-level efficacy attribution is often incomplete, and durability remains less certain than with the best-characterised autologous CD19 datasets. For now, the main conclusion is feasibility, not definitive superiority.

To support future trial design, **Table 4** summarises a hypothesis-generating comparison of platform categories. Autologous CD19 CAR-T currently has the most mature SLE-specific evidence and the strongest immune-reset narrative. BCMA-only or BCMA-containing products may be attractive for nephritis-rich, autoantibody-driven phenotypes but require longer follow-up of immunoglobulin recovery and infection risk. CD19/BCMA compound products offer a mechanistically broad strategy but require controlled comparison to determine whether broader targeting improves durability enough to justify any additional immune cost. Allogeneic CAR-T and CAR-NK platforms may improve access and speed but require careful durability, rejection, persistence and graft-versus-host or host-versus-product monitoring. *In vivo* CAR-T approaches could simplify delivery but raise distinct questions about biodistribution, dose control, reversibility and long-term vector safety (23, 61). None of these platform differences can be resolved by the current uncontrolled dataset.

**Table 4:** Hypothesis-generating comparison of CAR-based platform categories relevant to refractory SLE.

Platform or target strategy	Biological rationale	Current SLE evidence in this review	Key unresolved questions
Autologous CD19 CAR-T	Targets most B-lineage cells from early B cells through mature B cells and many plasmablasts; may reset antigen presentation, cytokine production and autoreactive B-cell repertoires.	Largest and most mature SLE-specific clinical experience; strong response and drug-free remission signals amongst exactly evaluable rows.	Durability beyond B-cell reconstitution; relapse mechanisms; infection and hypogammaglobulinaemia risk; access and manufacturing time.
BCMA-only or BCMA-containing CAR-T	Targets plasmablasts and plasma cells through a plasma-cell survival receptor embedded in BAFF/APRIL biology.	Encouraging renal-dominant and antibody-driven disease signals; exact patient-level data remain smaller than for CD19.	Whether broader plasma-cell targeting improves durability; impact on protective humoral immunity; immunoglobulin replacement needs.
CD19/BCMA compound or co-infused CAR-T	Combines upstream B-lineage depletion with downstream plasma-cell compartment targeting.	Promising phase 1 signals in refractory SLE and lupus nephritis-rich cohorts.	Whether dual targeting is superior to CD19 alone; optimal dosing; additive infection or cytopenia risk.
CD19/CD22 CAR-T	Broadens B-lineage targeting and may reduce single-antigen escape or incomplete B-cell depletion.	Very small paediatric lupus nephritis experience with response but notable inflammatory toxicity.	Paediatric safety; CRS/HLH/TMA risk; target burden and dose optimisation.
Allogeneic CD19 CAR-T	Off-the-shelf access may reduce manufacturing delay and dependence on patient T-cell fitness.	Early SLE cohorts show feasibility, B-cell depletion and encouraging short-term responses with no exact GVHD signal in evaluable rows.	Persistence, rejection, durability, GVHD risk, host-versus-product immunity and repeat dosing.
Allogeneic CD19 CAR-NK	NK-cell platforms may offer off-the-shelf dosing and potentially lower acute CRS/ICANS risk.	Largest CAR-NK SLE cohort uses mainly cohort-level reporting; patient-level efficacy attribution remains limited.	Durability, persistence, repeat infusion schedules, exact patient-level response and delayed infection risk.
*In vivo* CD19 CAR-T	Generates CAR-T cells inside the patient, potentially simplifying logistics and avoiding ex vivo manufacturing.	Early SLE letter-level experience suggests feasibility but remains very preliminary.	Dose control, biodistribution, reversibility, vector safety, long-term persistence and regulatory monitoring.
Emerging BAFF-axis, antigen-restricted or plasma-cell targets	BAFF-R/TACI/APRIL, IGHV4–34 and plasma-cell targets such as CD38 aim for more pathway-specific or compartment-specific immune editing.	Currently adjacent, preclinical or very early translational relative to counted CD19/BCMA SLE datasets.	Direct SLE efficacy, specificity, preservation of protective immunity, infection risk and how to integrate with existing biologics.

This table is intended to support translational interpretation and future trial design. It should not be interpreted as a formal comparative-efficacy analysis because the current evidence base is uncontrolled and endpoint completeness differs across platforms.

The relevance of CAR-based immune reset probably extends beyond SLE. CD19 CAR-T therapy has now shown encouraging results not only in lupus but also in systemic sclerosis, inflammatory myopathy, antisynthetase syndrome, and broader autoimmune cohorts (19, 40, 41, 62–67). These adjacent diseases matter because they reinforce the idea that deep depletion of autoreactive B-cell compartments might operate at a pathogenic-programme level rather than within a single nosological boundary (14, 15, 19, 40, 41). This has practical implications for trial design. The next generation of studies may increasingly use basket or platform frameworks, especially where severe disease is rare and organ-threatening phenotypes overlap mechanistically across connective tissue diseases. For lupus investigators, this broader context is a strength, but it also raises the bar for disease-specific inference: SLE-specific claims should still be anchored in SLE-specific data. Preclinical lupus data also support the disease-specific rationale for CD19-directed cellular therapy, as sustained B-cell depletion by CD19-targeted CAR T cells was shown to be highly effective in murine lupus (68).

A major strength of the present review is methodological. The SLE CAR literature is unusually vulnerable to distorted synthesis because the same therapeutic experience may appear as an index case, an expanded cohort, a longer follow-up paper, or an organ-specific companion report (15, 19, 27, 28). A conventional publication-level review can therefore overcount patients and overstate certainty. Our overlap-controlled approach, based on independent study units and a deduplicated patient-level master dataset, was intended to address exactly this problem. Equally important, we separated outcome prevalence from endpoint evaluability. In this field, many studies report outcomes at cohort level without sufficient patient-level mapping. Treating those studies as though every event were individually attributable would create pseudo-precision. The conservative exact-attribution framework used here is deliberately stricter and therefore more credible, even if it yields smaller denominators and more modest-looking prevalence estimates (26, 31).

Overlap control, however, does not solve publication bias or selective outcome reporting. In an emerging field dominated by single-patient reports, small case series, early-phase trials and conference abstracts, successful remissions and novel platforms are more likely to be reported rapidly than treatment failures, manufacturing failures, delayed infections or incomplete responses (33, 34). Selective reporting can also operate within published studies when remission, drug withdrawal or serological normalisation is highlighted whilst infection surveillance, immunoglobulin kinetics, B-cell reconstitution, late cytopenias or patient-level nonresponse is reported incompletely. For this reason, high response proportions amongst exactly evaluable rows should be read as evidence of a strong signal in selected reported patients, not as unbiased estimates of absolute response rates in all treated patients.

This review also has clear limitations. Most included evidence was uncontrolled; reporting was heterogeneous; follow-up remained limited; and patient-level endpoint completeness varied sharply across products and study units. Exact denominators were particularly sparse for renal improvement, DORIS remission, severe infection and special inflammatory toxicity, making those proportions unstable. The geographic concentration of the literature, particularly in China and Germany, may also affect external generalisability. Some included evidence came from conference-level or otherwise atypical records retained for completeness. Finally, formal between-platform comparison is premature (15, 20–25, 27, 28, 41, 42). These limitations do not undermine the central conclusion, but they should shape how the literature is read: the current data are compelling enough to justify prospective development but not yet mature enough to support strong comparative claims.

The immediate agenda for the field is therefore methodological discipline. Future studies should prospectively define remission, low disease activity, renal response, drug-free remission and immune reconstitution; use harmonised ASTCT toxicity reporting; distinguish CRS, ICANS, IEC-HS, severe infection, hypogammaglobulinaemia and late cytopenia explicitly; and capture B-cell and plasma-cell reconstitution phenotypes in a standardised way (7–12, 30, 43, 51–56). In lupus nephritis, histological chronicity and organ-specific endpoints should be incorporated more systematically. Comparative questions are now apparent: CD19 alone versus BCMA-containing constructs, CD19/BCMA compound versus sequential or single-target strategies, autologous versus allogeneic platforms, CAR-T versus CAR-NK, ex vivo versus *in vivo* engineering, and rescue-line versus earlier deployment. Based on the current evidence, CAR-based cellular therapy should be viewed as one of the most promising emerging strategies for true immune reset in refractory SLE, but its next phase must be built on better denominators, tighter endpoint definitions, long-term immune-safety monitoring and prospective controlled designs.

## Data Availability

The data analyzed in this systematic review were extracted from publicly available published reports and conference abstracts cited in the article. No dedicated public repository or accession number applies. The curated extraction workbook and figure-ready datasets are available from the corresponding author upon reasonable request.
